# Bis(2,2′-bipyridine-κ^2^ *N*,*N*′)(4-methyl­benzoato-κ^2^ *O*,*O*′)copper(II) iodide hemihydrate

**DOI:** 10.1107/S1600536808024252

**Published:** 2008-08-06

**Authors:** Chuan-Mei Tang, Guo-Hua Deng

**Affiliations:** a College of Chemistry, South China University of Technology, Guangzhou 510640, People’s Republic of China

## Abstract

The title compound, [Cu(C_8_H_7_O_2_)(C_10_H_8_N_2_)_2_]I·0.5H_2_O, was obtained by the hydro­thermal reaction of copper(I) iodide, 4-methyl­benzoic acid and 2,2′-bipyridine. The initial reactant of Cu^I^ was oxidized to Cu^II^. The asymmetric unit contains two independent complex mol­ecules, two I^−^ ions and one water molecule. Each Cu^II^ atom is coordinated by two O atoms from a 4-methyl­benzoate ligand and four N atoms from two 2,2′-bipyridine ligands, displaying a distorted octa­hedral geometry. The structure involves O—H⋯I hydrogen bonds between the water mol­ecule and iodide ions and π–π stacking inter­actions between the benzene and pyridyl rings [centroid–centroid distance = 3.79 (1) Å] and between the pyridyl rings [centroid–centroid distance = 3.87 (1) Å].

## Related literature

For related literature, see: Ma & Deng (2008 ▶); Mao *et al.* (2001 ▶); Song *et al.* (2008*a* ▶,*b* ▶,*c* ▶,*d* ▶).
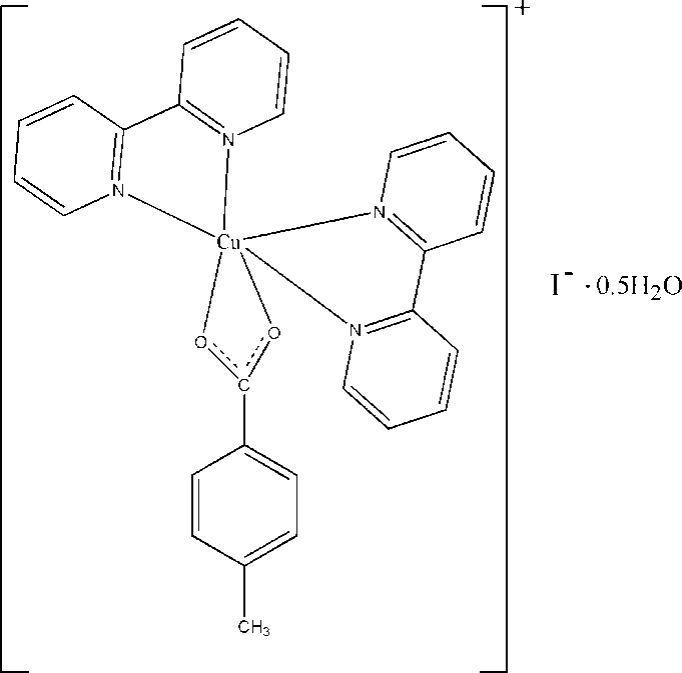


## Experimental

### 

#### Crystal data

[Cu(C_8_H_7_O_2_)(C_10_H_8_N_2_)_2_]I·0.5H_2_O
*M* *_r_* = 646.96
Triclinic, 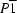

*a* = 14.6698 (4) Å
*b* = 15.3588 (4) Å
*c* = 15.4224 (7) Å
α = 100.943 (2)°
β = 114.345 (2)°
γ = 111.996 (2)°
*V* = 2680.64 (19) Å^3^
*Z* = 4
Mo *K*α radiation
μ = 2.00 mm^−1^
*T* = 296 (2) K
0.37 × 0.30 × 0.26 mm

#### Data collection

Bruker SMART APEXII CCD area-detector diffractometer
Absorption correction: multi-scan (*SADABS*; Sheldrick, 1996 ▶) *T*_min_ = 0.525, *T*_max_ = 0.624
29909 measured reflections
9564 independent reflections
6740 reflections with *I* > 2σ(*I*)
*R*_int_ = 0.037

#### Refinement

*R*[*F* ^2^ > 2σ(*F* ^2^)] = 0.046
*wR*(*F* ^2^) = 0.149
*S* = 1.03
9564 reflections
660 parameters
3 restraints
H-atom parameters constrained
Δρ_max_ = 1.55 e Å^−3^
Δρ_min_ = −1.53 e Å^−3^

### 

Data collection: *APEX2* (Bruker, 2007 ▶); cell refinement: *SAINT* (Bruker, 2007 ▶); data reduction: *SAINT*; program(s) used to solve structure: *SHELXS97* (Sheldrick, 2008 ▶); program(s) used to refine structure: *SHELXL97* (Sheldrick, 2008 ▶); molecular graphics: *SHELXTL* (Sheldrick, 2008 ▶); software used to prepare material for publication: *SHELXTL*.

## Supplementary Material

Crystal structure: contains datablocks I, global. DOI: 10.1107/S1600536808024252/hy2148sup1.cif

Structure factors: contains datablocks I. DOI: 10.1107/S1600536808024252/hy2148Isup2.hkl

Additional supplementary materials:  crystallographic information; 3D view; checkCIF report

## Figures and Tables

**Table 1:** Selected bond lengths (Å)

Cu1—O1	1.976 (4)
Cu1—O2	2.769 (4)
Cu1—N6	1.987 (4)
Cu1—N3	2.000 (4)
Cu1—N5	2.060 (4)
Cu1—N4	2.192 (4)
Cu2—O3	1.974 (3)
Cu2—O4	2.832 (3)
Cu2—N8	1.997 (4)
Cu2—N2	2.001 (4)
Cu2—N7	2.038 (4)
Cu2—N1	2.181 (4)

**Table 2:** Hydrogen-bond geometry (Å, °)

*D*—H⋯*A*	*D*—H	H⋯*A*	*D*⋯*A*	*D*—H⋯*A*
O1*W*—H1*W*⋯I1^i^	0.82	3.15	3.935 (8)	161
O1*W*—H2*W*⋯I1^ii^	0.82	2.76	3.568 (8)	170

Symmetry codes: (i) 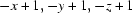; (ii) 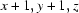.

**Fig. 1.**
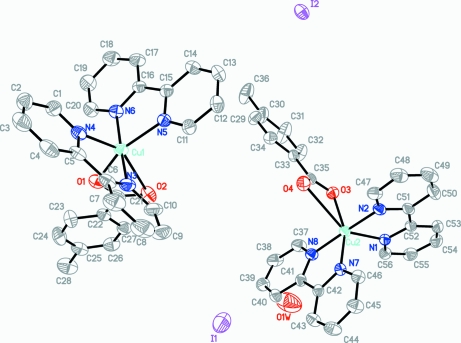
The molecular structure of the title compound. Displacement ellipsoids are drawn at the 30% probability level. H atoms have been omitted for clarity.

**Fig. 2.**
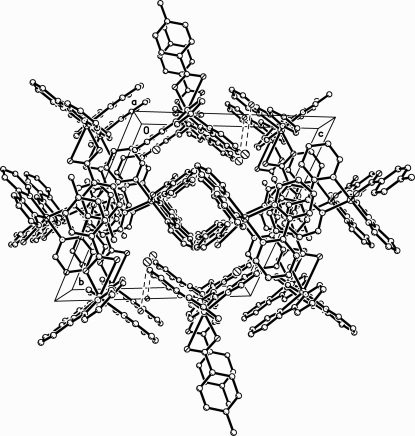
A packing view of the title compound. Hydrogen bonds are depicted as dashed lines.

## supplementary crystallographic information

### Comment

As a building block, 4-methylbenzoate ligand is an excellent candidate for the construction of supramolecular complexes (Ma & Deng, 2008; Song *et al.*, 2008*a*,b,c,d). Recently, we obtained the title mononuclear complex by the hydrothermal reaction of CuI, 4-methylbenzoic acid and 2,2'-bipyridine.

As illustrated in Fig. 1, the asymmetric unit of the title compound contains two independent complex molecules, two I ions and one lattice water molecule. Each Cu^II^ atom has a distorted octahedral coordination geometry, involving two carboxylate O atoms from one 4-methylbenzoate ligand, and four N atoms from two 2,2'-bipyridine ligands. One Cu—O distance is distinctly longer than the others for each Cu^II^ atom (Table 1), but still within the range of a significant interaction (Mao *et al.* 2001). Judged from the blue crystals, the initial reactant of Cu^I^ was thus oxidated to Cu^II^ in the hydrothermal reaction. The structure involves O—H···I hydrogen bonds between the water molecule and I ions (Table 2) and π–π stacking interactions (Fig. 2). The centroid–centroid distances are 3.79 (1)Å between the adjacent phenyl and pyridyl rings, and 3.87 (1)Å between the adjacent pyridyl rings.

### Experimental

A mixture of CuI (0.1 g, 0.5 mmol), 4-methylbenzoic acid (0.068 g, 0.5 mmol), 2,2'-bipyridine (0.078 g, 0.5 mmol) and H_2_O (10 ml) was placed in a 23 ml Teflon-lined reactor, which was heated to 433 K for 3 d and then cooled to room temperature at a rate of 10 K h^-1^. Block colorless crystals were obtained.

### Refinement

C-bound H atoms were positioned geometrically and refined as riding, with C—H = 0.93 (CH) and 0.96 (CH_3_) Å, and with *U*_iso_(H) = 1.2*U*_eq_(C) for CH group or 1.5*U*_eq_(C) for CH_3_ group. H atoms of water molecule were tentatively located in difference Fourier maps and refined with distance restraints of O–H = 0.84 (1) and H···H = 1.35 (1) Å, and with *U*_iso_(H) = 1.5*U*_eq_(O). The highest residual electron density was 1.14 Å from atom I1 and the deepest hole 0.81 Å from atom I1.

### Crystal data

**Table:**

[Cu(C_8_H_7_O_2_)(C_10_H_8_N_2_)_2_]I·0.5H_2_O	*Z* = 4
*M**_r_* = 646.96	*F*_000_ = 1288
Triclinic, *P*1	*D*_x_ = 1.603 Mg m^−^^3^
Hall symbol: -P 1	Mo *K*α radiation λ = 0.71073 Å
*a* = 14.6698 (4) Å	Cell parameters from 5300 reflections
*b* = 15.3588 (4) Å	θ = 1.3–28.0º
*c* = 15.4224 (7) Å	µ = 2.00 mm^−^^1^
α = 100.943 (2)º	*T* = 296 (2) K
β = 114.345 (2)º	Block, blue
γ = 111.996 (2)º	0.37 × 0.30 × 0.26 mm
*V* = 2680.64 (19) Å^3^	

### Data collection

**Table:**

Bruker SMART APEXII CCD area-detector diffractometer	9564 independent reflections
Radiation source: fine-focus sealed tube	6740 reflections with *I* > 2σ(*I*)
Monochromator: graphite	*R*_int_ = 0.037
*T* = 296(2) K	θ_max_ = 25.2º
φ and ω scans	θ_min_ = 1.6º
Absorption correction: multi-scan(SADABS; Sheldrick, 1996)	*h* = −17→17
*T*_min_ = 0.525, *T*_max_ = 0.624	*k* = −18→18
29909 measured reflections	*l* = −17→18

### Refinement

**Table:**

Refinement on *F*^2^	Secondary atom site location: difference Fourier map
Least-squares matrix: full	Hydrogen site location: inferred from neighbouring sites
*R*[*F*^2^ > 2σ(*F*^2^)] = 0.047	H-atom parameters constrained
*wR*(*F*^2^) = 0.149	*w* = 1/[σ^2^(*F*_o_^2^) + (0.0774*P*)^2^ + 2.4876*P*] where *P* = (*F*_o_^2^ + 2*F*_c_^2^)/3
*S* = 1.03	(Δ/σ)_max_ < 0.001
9564 reflections	Δρ_max_ = 1.56 e Å^−^^3^
660 parameters	Δρ_min_ = −1.53 e Å^−^^3^
3 restraints	Extinction correction: none
Primary atom site location: structure-invariant direct methods	

### Fractional atomic coordinates and isotropic or equivalent isotropic displacement parameters (Å^2^)

**Table:**

	*x*	*y*	*z*	*U*_iso_*/*U*_eq_	
C1	0.5583 (5)	−0.0992 (4)	0.8879 (4)	0.0588 (14)	
H1	0.6232	−0.0884	0.8844	0.071*	
C2	0.5284 (6)	−0.1583 (5)	0.9396 (5)	0.0685 (16)	
H2	0.5726	−0.1862	0.9707	0.082*	
C3	0.4332 (6)	−0.1746 (5)	0.9441 (5)	0.0787 (19)	
H3	0.4115	−0.2137	0.9787	0.094*	
C4	0.3693 (5)	−0.1328 (5)	0.8973 (5)	0.0698 (17)	
H4	0.3035	−0.1439	0.8992	0.084*	
C5	0.4036 (4)	−0.0738 (4)	0.8470 (4)	0.0515 (12)	
C6	0.3422 (4)	−0.0237 (4)	0.7958 (4)	0.0543 (13)	
C7	0.2355 (6)	−0.0438 (5)	0.7799 (6)	0.083 (2)	
H7	0.1976	−0.0916	0.7998	0.099*	
C8	0.1861 (6)	0.0083 (7)	0.7338 (7)	0.100 (3)	
H8	0.1139	−0.0047	0.7221	0.121*	
C9	0.2416 (7)	0.0782 (7)	0.7056 (6)	0.091 (2)	
H9	0.2095	0.1151	0.6767	0.110*	
C10	0.3481 (5)	0.0941 (5)	0.7206 (5)	0.0719 (17)	
H10	0.3862	0.1408	0.6997	0.086*	
C11	0.3340 (4)	−0.0838 (4)	0.5409 (4)	0.0536 (13)	
H11	0.2900	−0.0645	0.5605	0.064*	
C12	0.2824 (5)	−0.1491 (4)	0.4398 (4)	0.0617 (15)	
H12	0.2052	−0.1726	0.3913	0.074*	
C13	0.3469 (5)	−0.1793 (4)	0.4111 (4)	0.0632 (15)	
H13	0.3130	−0.2250	0.3433	0.076*	
C14	0.4609 (5)	−0.1414 (4)	0.4830 (4)	0.0573 (13)	
H14	0.5056	−0.1600	0.4640	0.069*	
C15	0.5095 (4)	−0.0754 (3)	0.5840 (4)	0.0441 (11)	
C16	0.6312 (4)	−0.0306 (3)	0.6684 (4)	0.0443 (11)	
C17	0.7116 (5)	−0.0489 (4)	0.6564 (5)	0.0617 (14)	
H17	0.6910	−0.0899	0.5912	0.074*	
C18	0.8221 (5)	−0.0065 (5)	0.7407 (5)	0.0733 (17)	
H18	0.8765	−0.0189	0.7334	0.088*	
C19	0.8507 (5)	0.0547 (5)	0.8365 (5)	0.0703 (16)	
H19	0.9242	0.0830	0.8950	0.084*	
C20	0.7693 (4)	0.0734 (4)	0.8442 (4)	0.0595 (14)	
H20	0.7894	0.1163	0.9083	0.071*	
C21	0.6656 (4)	0.2547 (4)	0.8568 (4)	0.0494 (12)	
C22	0.7626 (4)	0.3594 (4)	0.9370 (4)	0.0472 (12)	
C23	0.8244 (5)	0.3793 (5)	1.0407 (4)	0.0666 (16)	
H23	0.8066	0.3261	1.0623	0.080*	
C24	0.9126 (5)	0.4775 (5)	1.1130 (5)	0.0778 (18)	
H24	0.9520	0.4891	1.1829	0.093*	
C25	0.9441 (5)	0.5588 (4)	1.0854 (5)	0.0642 (16)	
C26	0.8836 (5)	0.5382 (4)	0.9817 (5)	0.0664 (16)	
H26	0.9037	0.5911	0.9600	0.080*	
C27	0.7936 (5)	0.4407 (4)	0.9084 (4)	0.0577 (13)	
H27	0.7531	0.4297	0.8387	0.069*	
C28	1.0395 (6)	0.6647 (5)	1.1648 (6)	0.099 (2)	
H28A	1.0074	0.7023	1.1859	0.148*	
H28B	1.0801	0.6992	1.1353	0.148*	
H28C	1.0924	0.6605	1.2243	0.148*	
C36	1.0037 (6)	0.3445 (7)	0.4202 (7)	0.105 (3)	
H36A	0.9998	0.3090	0.3592	0.158*	
H36B	1.0105	0.3080	0.4644	0.158*	
H36C	1.0701	0.4127	0.4573	0.158*	
C30	0.8952 (5)	0.3506 (5)	0.3882 (5)	0.0709 (17)	
C31	0.8731 (6)	0.4128 (7)	0.3403 (6)	0.098 (3)	
H31	0.9283	0.4540	0.3288	0.118*	
C32	0.7725 (5)	0.4176 (5)	0.3080 (5)	0.0744 (18)	
H32	0.7607	0.4606	0.2751	0.089*	
C33	0.6900 (4)	0.3575 (4)	0.3255 (4)	0.0432 (11)	
C34	0.7122 (4)	0.2956 (4)	0.3758 (4)	0.0521 (12)	
H34	0.6580	0.2551	0.3886	0.063*	
C35	0.5819 (4)	0.3622 (4)	0.2935 (4)	0.0461 (11)	
C37	0.6852 (4)	0.6225 (4)	0.4203 (5)	0.0648 (15)	
H37	0.7121	0.6057	0.3783	0.078*	
C38	0.7636 (5)	0.6975 (4)	0.5194 (5)	0.0746 (18)	
H38	0.8424	0.7318	0.5438	0.090*	
C39	0.7249 (5)	0.7213 (4)	0.5815 (5)	0.0669 (16)	
H39	0.7775	0.7697	0.6501	0.080*	
C40	0.6076 (5)	0.6734 (4)	0.5428 (4)	0.0562 (13)	
H40	0.5798	0.6901	0.5840	0.067*	
C41	0.5320 (4)	0.6000 (3)	0.4412 (4)	0.0441 (11)	
C42	0.4036 (4)	0.5452 (3)	0.3899 (3)	0.0417 (11)	
C43	0.3480 (5)	0.5727 (4)	0.4316 (4)	0.0569 (13)	
H43	0.3898	0.6275	0.4960	0.068*	
C44	0.2272 (5)	0.5173 (5)	0.3759 (5)	0.0681 (16)	
H44	0.1867	0.5358	0.4015	0.082*	
C45	0.1691 (5)	0.4352 (4)	0.2830 (5)	0.0627 (15)	
H45	0.0886	0.3955	0.2457	0.075*	
C46	0.2306 (4)	0.4124 (4)	0.2458 (4)	0.0506 (12)	
H46	0.1902	0.3567	0.1824	0.061*	
C47	0.2668 (5)	0.2356 (4)	0.1058 (5)	0.0598 (14)	
H47	0.2849	0.2271	0.1678	0.072*	
C48	0.1841 (5)	0.1508 (4)	0.0136 (6)	0.0715 (17)	
H48	0.1474	0.0861	0.0126	0.086*	
C49	0.1574 (5)	0.1646 (5)	−0.0771 (6)	0.082 (2)	
H49	0.1013	0.1087	−0.1408	0.098*	
C50	0.2138 (5)	0.2621 (5)	−0.0742 (4)	0.0706 (17)	
H50	0.1966	0.2717	−0.1356	0.085*	
C51	0.2964 (4)	0.3448 (4)	0.0223 (4)	0.0499 (12)	
C52	0.3624 (4)	0.4512 (4)	0.0340 (4)	0.0481 (12)	
C29	0.8130 (5)	0.2930 (4)	0.4069 (5)	0.0606 (14)	
H29	0.8261	0.2514	0.4414	0.073*	
C53	0.3479 (5)	0.4781 (5)	−0.0497 (4)	0.0609 (14)	
H53	0.2921	0.4294	−0.1177	0.073*	
C54	0.4170 (6)	0.5774 (5)	−0.0305 (5)	0.0700 (17)	
H54	0.4096	0.5968	−0.0856	0.084*	
C55	0.4974 (5)	0.6486 (5)	0.0704 (5)	0.0643 (15)	
H55	0.5449	0.7165	0.0847	0.077*	
C56	0.5062 (5)	0.6173 (4)	0.1499 (4)	0.0533 (13)	
H56	0.5604	0.6655	0.2183	0.064*	
Cu2	0.44994 (5)	0.45165 (4)	0.24535 (4)	0.04330 (16)	
Cu1	0.53604 (5)	0.04754 (4)	0.76873 (5)	0.04622 (17)	
I1	0.04839 (6)	0.20792 (5)	0.62512 (4)	0.1112 (2)	
I2	0.98058 (3)	0.14574 (3)	0.13668 (3)	0.07583 (16)	
N1	0.4398 (3)	0.5204 (3)	0.1327 (3)	0.0449 (9)	
N2	0.3228 (3)	0.3298 (3)	0.1110 (3)	0.0483 (10)	
N3	0.3964 (4)	0.0430 (3)	0.7649 (3)	0.0544 (11)	
N4	0.4981 (3)	−0.0577 (3)	0.8433 (3)	0.0498 (10)	
N5	0.4459 (3)	−0.0467 (3)	0.6130 (3)	0.0440 (9)	
N6	0.6613 (3)	0.0313 (3)	0.7616 (3)	0.0479 (10)	
N7	0.3464 (3)	0.4665 (3)	0.2964 (3)	0.0416 (9)	
N8	0.5707 (3)	0.5723 (3)	0.3818 (3)	0.0478 (10)	
O1	0.6478 (3)	0.1823 (3)	0.8889 (3)	0.0568 (9)	
O2	0.6079 (3)	0.2405 (3)	0.7651 (3)	0.0674 (10)	
O3	0.5675 (3)	0.4192 (3)	0.2437 (3)	0.0522 (8)	
O4	0.5122 (3)	0.3137 (3)	0.3162 (3)	0.0597 (9)	
O1W	0.8745 (9)	0.9386 (6)	0.5468 (7)	0.201 (4)	
H2W	0.9082	1.0013	0.5663	0.301*	
H1W	0.8900	0.9191	0.5042	0.301*	

### Atomic displacement parameters (Å^2^)

**Table:**

	*U*^11^	*U*^22^	*U*^33^	*U*^12^	*U*^13^	*U*^23^
C1	0.063 (3)	0.062 (3)	0.066 (4)	0.034 (3)	0.041 (3)	0.030 (3)
C2	0.075 (4)	0.066 (4)	0.066 (4)	0.034 (3)	0.039 (3)	0.031 (3)
C3	0.093 (5)	0.070 (4)	0.078 (4)	0.029 (4)	0.062 (4)	0.028 (3)
C4	0.069 (4)	0.064 (4)	0.067 (4)	0.018 (3)	0.050 (3)	0.011 (3)
C5	0.049 (3)	0.045 (3)	0.048 (3)	0.015 (2)	0.031 (3)	0.004 (2)
C6	0.042 (3)	0.056 (3)	0.049 (3)	0.017 (2)	0.027 (3)	0.001 (2)
C7	0.058 (4)	0.090 (5)	0.097 (5)	0.035 (4)	0.049 (4)	0.020 (4)
C8	0.060 (4)	0.124 (7)	0.101 (6)	0.050 (5)	0.041 (4)	0.011 (5)
C9	0.079 (5)	0.106 (6)	0.091 (5)	0.071 (5)	0.034 (4)	0.023 (4)
C10	0.067 (4)	0.069 (4)	0.076 (4)	0.046 (3)	0.032 (3)	0.013 (3)
C11	0.049 (3)	0.054 (3)	0.048 (3)	0.023 (2)	0.022 (3)	0.018 (2)
C12	0.051 (3)	0.065 (4)	0.045 (3)	0.021 (3)	0.016 (3)	0.017 (3)
C13	0.068 (4)	0.068 (4)	0.043 (3)	0.028 (3)	0.029 (3)	0.015 (3)
C14	0.065 (4)	0.055 (3)	0.056 (3)	0.030 (3)	0.037 (3)	0.017 (3)
C15	0.047 (3)	0.040 (2)	0.050 (3)	0.021 (2)	0.029 (2)	0.022 (2)
C16	0.048 (3)	0.043 (3)	0.053 (3)	0.027 (2)	0.032 (2)	0.022 (2)
C17	0.059 (3)	0.062 (3)	0.070 (4)	0.034 (3)	0.038 (3)	0.019 (3)
C18	0.059 (4)	0.083 (4)	0.087 (5)	0.043 (3)	0.042 (4)	0.029 (4)
C19	0.042 (3)	0.085 (4)	0.070 (4)	0.030 (3)	0.025 (3)	0.021 (3)
C20	0.041 (3)	0.065 (3)	0.055 (3)	0.022 (3)	0.022 (3)	0.015 (3)
C21	0.044 (3)	0.054 (3)	0.048 (3)	0.022 (2)	0.031 (3)	0.009 (2)
C22	0.039 (3)	0.043 (3)	0.049 (3)	0.016 (2)	0.023 (2)	0.008 (2)
C23	0.060 (4)	0.069 (4)	0.052 (3)	0.019 (3)	0.027 (3)	0.023 (3)
C24	0.056 (4)	0.084 (5)	0.047 (3)	0.017 (3)	0.014 (3)	0.010 (3)
C25	0.041 (3)	0.056 (3)	0.067 (4)	0.015 (3)	0.026 (3)	0.000 (3)
C26	0.054 (3)	0.049 (3)	0.083 (5)	0.021 (3)	0.034 (3)	0.019 (3)
C27	0.055 (3)	0.054 (3)	0.056 (3)	0.027 (3)	0.026 (3)	0.016 (3)
C28	0.057 (4)	0.075 (4)	0.098 (5)	0.013 (3)	0.026 (4)	−0.008 (4)
C36	0.064 (4)	0.188 (8)	0.124 (6)	0.083 (5)	0.063 (5)	0.107 (7)
C30	0.055 (3)	0.109 (5)	0.072 (4)	0.048 (3)	0.039 (3)	0.053 (4)
C31	0.059 (4)	0.168 (7)	0.121 (6)	0.064 (5)	0.062 (4)	0.109 (6)
C32	0.057 (4)	0.097 (5)	0.088 (5)	0.041 (3)	0.041 (3)	0.061 (4)
C33	0.038 (3)	0.052 (3)	0.039 (3)	0.022 (2)	0.021 (2)	0.016 (2)
C34	0.050 (3)	0.056 (3)	0.060 (3)	0.027 (2)	0.037 (3)	0.025 (3)
C35	0.042 (3)	0.055 (3)	0.031 (2)	0.025 (2)	0.015 (2)	0.006 (2)
C37	0.041 (3)	0.061 (3)	0.071 (4)	0.022 (3)	0.025 (3)	0.007 (3)
C38	0.036 (3)	0.057 (3)	0.088 (5)	0.020 (3)	0.015 (3)	0.000 (3)
C39	0.051 (3)	0.053 (3)	0.058 (3)	0.023 (3)	0.009 (3)	0.003 (3)
C40	0.053 (3)	0.055 (3)	0.044 (3)	0.024 (3)	0.019 (3)	0.010 (2)
C41	0.042 (3)	0.039 (2)	0.039 (3)	0.017 (2)	0.015 (2)	0.015 (2)
C42	0.042 (3)	0.039 (2)	0.039 (3)	0.018 (2)	0.020 (2)	0.017 (2)
C43	0.056 (3)	0.053 (3)	0.054 (3)	0.021 (3)	0.033 (3)	0.014 (2)
C44	0.070 (4)	0.066 (4)	0.080 (4)	0.032 (3)	0.054 (4)	0.023 (3)
C45	0.048 (3)	0.060 (3)	0.075 (4)	0.021 (3)	0.038 (3)	0.018 (3)
C46	0.042 (3)	0.045 (3)	0.053 (3)	0.015 (2)	0.023 (2)	0.014 (2)
C47	0.051 (3)	0.051 (3)	0.067 (4)	0.026 (3)	0.029 (3)	0.013 (3)
C48	0.051 (3)	0.050 (3)	0.084 (5)	0.015 (3)	0.032 (3)	0.005 (3)
C49	0.044 (3)	0.067 (4)	0.077 (5)	0.011 (3)	0.020 (3)	−0.015 (3)
C50	0.049 (3)	0.087 (5)	0.045 (3)	0.026 (3)	0.019 (3)	0.003 (3)
C51	0.040 (3)	0.061 (3)	0.041 (3)	0.027 (2)	0.020 (2)	0.007 (2)
C52	0.044 (3)	0.064 (3)	0.043 (3)	0.035 (2)	0.025 (2)	0.016 (2)
C29	0.062 (3)	0.076 (4)	0.070 (4)	0.045 (3)	0.041 (3)	0.043 (3)
C53	0.067 (4)	0.084 (4)	0.044 (3)	0.049 (3)	0.029 (3)	0.026 (3)
C54	0.092 (5)	0.094 (5)	0.062 (4)	0.066 (4)	0.048 (4)	0.046 (4)
C55	0.081 (4)	0.067 (4)	0.075 (4)	0.046 (3)	0.052 (4)	0.041 (3)
C56	0.062 (3)	0.053 (3)	0.053 (3)	0.034 (3)	0.032 (3)	0.025 (3)
Cu2	0.0401 (3)	0.0434 (3)	0.0389 (3)	0.0195 (3)	0.0192 (3)	0.0103 (2)
Cu1	0.0409 (3)	0.0460 (3)	0.0491 (4)	0.0218 (3)	0.0244 (3)	0.0136 (3)
I1	0.1320 (5)	0.1664 (6)	0.0966 (4)	0.1121 (5)	0.0670 (4)	0.0762 (4)
I2	0.0619 (3)	0.0608 (3)	0.0672 (3)	0.01033 (19)	0.0296 (2)	0.0105 (2)
N1	0.045 (2)	0.053 (2)	0.040 (2)	0.029 (2)	0.0223 (19)	0.0163 (18)
N2	0.042 (2)	0.043 (2)	0.046 (2)	0.0190 (18)	0.0195 (19)	0.0059 (18)
N3	0.046 (2)	0.055 (3)	0.054 (3)	0.028 (2)	0.024 (2)	0.009 (2)
N4	0.046 (2)	0.051 (2)	0.051 (2)	0.021 (2)	0.031 (2)	0.015 (2)
N5	0.044 (2)	0.039 (2)	0.045 (2)	0.0182 (18)	0.023 (2)	0.0153 (17)
N6	0.042 (2)	0.046 (2)	0.053 (3)	0.0201 (19)	0.027 (2)	0.017 (2)
N7	0.040 (2)	0.043 (2)	0.039 (2)	0.0192 (18)	0.0207 (18)	0.0167 (17)
N8	0.040 (2)	0.049 (2)	0.049 (2)	0.0215 (19)	0.023 (2)	0.0126 (19)
O1	0.058 (2)	0.046 (2)	0.059 (2)	0.0213 (17)	0.0337 (19)	0.0136 (17)
O2	0.068 (2)	0.065 (2)	0.048 (2)	0.022 (2)	0.029 (2)	0.0106 (18)
O3	0.053 (2)	0.060 (2)	0.051 (2)	0.0343 (17)	0.0282 (17)	0.0239 (17)
O4	0.048 (2)	0.084 (3)	0.058 (2)	0.0359 (19)	0.0337 (19)	0.031 (2)
O1W	0.325 (12)	0.176 (7)	0.233 (9)	0.142 (8)	0.225 (10)	0.116 (7)

### Geometric parameters (Å, °)

**Table:**

C1—N4	1.322 (6)	C31—H31	0.9300
C1—C2	1.384 (8)	C32—C33	1.385 (7)
C1—H1	0.9300	C32—H32	0.9300
C2—C3	1.357 (9)	C33—C34	1.384 (7)
C2—H2	0.9300	C33—C35	1.489 (6)
C3—C4	1.372 (9)	C34—C29	1.372 (7)
C3—H3	0.9300	C34—H34	0.9300
C4—C5	1.387 (8)	C35—O4	1.243 (6)
C4—H4	0.9300	C35—O3	1.289 (6)
C5—N4	1.341 (6)	C37—N8	1.342 (6)
C5—C6	1.480 (8)	C37—C38	1.370 (8)
C6—N3	1.340 (7)	C37—H37	0.9300
C6—C7	1.377 (8)	C38—C39	1.358 (9)
C7—C8	1.377 (11)	C38—H38	0.9300
C7—H7	0.9300	C39—C40	1.377 (8)
C8—C9	1.348 (11)	C39—H39	0.9300
C8—H8	0.9300	C40—C41	1.384 (7)
C9—C10	1.395 (9)	C40—H40	0.9300
C9—H9	0.9300	C41—N8	1.344 (6)
C10—N3	1.344 (7)	C41—C42	1.487 (6)
C10—H10	0.9300	C42—N7	1.351 (6)
C11—N5	1.346 (6)	C42—C43	1.358 (7)
C11—C12	1.369 (7)	C43—C44	1.393 (8)
C11—H11	0.9300	C43—H43	0.9300
C12—C13	1.380 (8)	C44—C45	1.368 (8)
C12—H12	0.9300	C44—H44	0.9300
C13—C14	1.364 (8)	C45—C46	1.360 (7)
C13—H13	0.9300	C45—H45	0.9300
C14—C15	1.380 (7)	C46—N7	1.338 (6)
C14—H14	0.9300	C46—H46	0.9300
C15—N5	1.357 (6)	C47—N2	1.336 (7)
C15—C16	1.476 (7)	C47—C48	1.371 (8)
C16—N6	1.345 (6)	C47—H47	0.9300
C16—C17	1.384 (7)	C48—C49	1.370 (10)
C17—C18	1.375 (8)	C48—H48	0.9300
C17—H17	0.9300	C49—C50	1.394 (9)
C18—C19	1.382 (9)	C49—H49	0.9300
C18—H18	0.9300	C50—C51	1.393 (7)
C19—C20	1.372 (8)	C50—H50	0.9300
C19—H19	0.9300	C51—N2	1.349 (6)
C20—N6	1.344 (6)	C51—C52	1.484 (7)
C20—H20	0.9300	C52—N1	1.339 (6)
C21—O2	1.233 (6)	C52—C53	1.387 (7)
C21—O1	1.284 (6)	C29—H29	0.9300
C21—C22	1.490 (7)	C53—C54	1.366 (8)
C22—C23	1.373 (7)	C53—H53	0.9300
C22—C27	1.381 (7)	C54—C55	1.372 (8)
C23—C24	1.380 (8)	C54—H54	0.9300
C23—H23	0.9300	C55—C56	1.375 (7)
C24—C25	1.373 (9)	C55—H55	0.9300
C24—H24	0.9300	C56—N1	1.336 (6)
C25—C26	1.369 (8)	C56—H56	0.9300
C25—C28	1.492 (8)	Cu1—O1	1.976 (4)
C26—C27	1.381 (8)	Cu1—O2	2.769 (4)
C26—H26	0.9300	Cu1—N6	1.987 (4)
C27—H27	0.9300	Cu1—N3	2.000 (4)
C28—H28A	0.9600	Cu1—N5	2.060 (4)
C28—H28B	0.9600	Cu1—N4	2.192 (4)
C28—H28C	0.9600	Cu2—O3	1.974 (3)
C36—C30	1.506 (8)	Cu2—O4	2.832 (3)
C36—H36A	0.9600	Cu2—N8	1.997 (4)
C36—H36B	0.9600	Cu2—N2	2.001 (4)
C36—H36C	0.9600	Cu2—N7	2.038 (4)
C30—C31	1.367 (8)	Cu2—N1	2.181 (4)
C30—C29	1.379 (8)	O1W—H2W	0.8200
C31—C32	1.385 (8)	O1W—H1W	0.8200
			
N4—C1—C2	122.5 (5)	C37—C38—H38	120.4
N4—C1—H1	118.7	C38—C39—C40	119.8 (5)
C2—C1—H1	118.7	C38—C39—H39	120.1
C3—C2—C1	118.7 (6)	C40—C39—H39	120.1
C3—C2—H2	120.6	C39—C40—C41	118.5 (5)
C1—C2—H2	120.6	C39—C40—H40	120.7
C2—C3—C4	119.3 (6)	C41—C40—H40	120.7
C2—C3—H3	120.3	N8—C41—C40	121.6 (5)
C4—C3—H3	120.3	N8—C41—C42	115.0 (4)
C3—C4—C5	119.5 (6)	C40—C41—C42	123.5 (5)
C3—C4—H4	120.2	N7—C42—C43	122.7 (4)
C5—C4—H4	120.2	N7—C42—C41	113.9 (4)
N4—C5—C4	120.7 (5)	C43—C42—C41	123.4 (4)
N4—C5—C6	115.5 (4)	C42—C43—C44	118.7 (5)
C4—C5—C6	123.8 (5)	C42—C43—H43	120.7
N3—C6—C7	121.3 (6)	C44—C43—H43	120.7
N3—C6—C5	116.0 (4)	C45—C44—C43	118.8 (5)
C7—C6—C5	122.8 (6)	C45—C44—H44	120.6
C6—C7—C8	118.7 (7)	C43—C44—H44	120.6
C6—C7—H7	120.7	C46—C45—C44	119.2 (5)
C8—C7—H7	120.7	C46—C45—H45	120.4
C9—C8—C7	120.6 (7)	C44—C45—H45	120.4
C9—C8—H8	119.7	N7—C46—C45	123.0 (5)
C7—C8—H8	119.7	N7—C46—H46	118.5
C8—C9—C10	118.8 (7)	C45—C46—H46	118.5
C8—C9—H9	120.6	N2—C47—C48	123.2 (6)
C10—C9—H9	120.6	N2—C47—H47	118.4
N3—C10—C9	120.8 (7)	C48—C47—H47	118.4
N3—C10—H10	119.6	C49—C48—C47	117.8 (6)
C9—C10—H10	119.6	C49—C48—H48	121.1
N5—C11—C12	122.4 (5)	C47—C48—H48	121.1
N5—C11—H11	118.8	C48—C49—C50	120.3 (6)
C12—C11—H11	118.8	C48—C49—H49	119.8
C11—C12—C13	118.8 (5)	C50—C49—H49	119.8
C11—C12—H12	120.6	C51—C50—C49	118.7 (6)
C13—C12—H12	120.6	C51—C50—H50	120.6
C14—C13—C12	119.5 (5)	C49—C50—H50	120.6
C14—C13—H13	120.3	N2—C51—C50	120.2 (5)
C12—C13—H13	120.3	N2—C51—C52	116.7 (4)
C13—C14—C15	119.7 (5)	C50—C51—C52	123.0 (5)
C13—C14—H14	120.1	N1—C52—C53	121.9 (5)
C15—C14—H14	120.1	N1—C52—C51	114.6 (4)
N5—C15—C14	121.0 (5)	C53—C52—C51	123.5 (5)
N5—C15—C16	114.4 (4)	C34—C29—C30	121.6 (5)
C14—C15—C16	124.6 (5)	C34—C29—H29	119.2
N6—C16—C17	120.4 (5)	C30—C29—H29	119.2
N6—C16—C15	115.3 (4)	C54—C53—C52	118.8 (5)
C17—C16—C15	124.3 (5)	C54—C53—H53	120.6
C18—C17—C16	120.1 (6)	C52—C53—H53	120.6
C18—C17—H17	119.9	C53—C54—C55	119.7 (5)
C16—C17—H17	119.9	C53—C54—H54	120.1
C17—C18—C19	118.7 (6)	C55—C54—H54	120.1
C17—C18—H18	120.7	C54—C55—C56	118.6 (6)
C19—C18—H18	120.7	C54—C55—H55	120.7
C20—C19—C18	119.3 (6)	C56—C55—H55	120.7
C20—C19—H19	120.4	N1—C56—C55	122.6 (5)
C18—C19—H19	120.4	N1—C56—H56	118.7
N6—C20—C19	121.7 (5)	C55—C56—H56	118.7
N6—C20—H20	119.1	O1—Cu1—O2	52.46 (16)
C19—C20—H20	119.1	O1—Cu1—N6	92.58 (16)
O2—C21—O1	123.2 (5)	O1—Cu1—N3	94.78 (16)
O2—C21—C22	120.5 (5)	O2—Cu1—N6	96.13 (16)
O1—C21—C22	116.3 (5)	O2—Cu1—N3	90.52 (16)
C23—C22—C27	117.5 (5)	N6—Cu1—N3	172.15 (16)
C23—C22—C21	122.2 (5)	O1—Cu1—N5	152.67 (15)
C27—C22—C21	120.3 (5)	O2—Cu1—N5	101.75 (16)
C22—C23—C24	120.7 (6)	N6—Cu1—N5	80.38 (16)
C22—C23—H23	119.7	N3—Cu1—N5	94.23 (16)
C24—C23—H23	119.7	O1—Cu1—N4	102.51 (15)
C25—C24—C23	122.3 (6)	O2—Cu1—N4	152.05 (16)
C25—C24—H24	118.9	N6—Cu1—N4	97.40 (16)
C23—C24—H24	118.9	N3—Cu1—N4	78.34 (17)
C26—C25—C24	116.8 (5)	N5—Cu1—N4	104.56 (15)
C26—C25—C28	121.7 (6)	O3—Cu2—O4	51.67 (15)
C24—C25—C28	121.6 (6)	O3—Cu2—N8	90.84 (15)
C25—C26—C27	121.7 (6)	O3—Cu2—N2	91.78 (15)
C25—C26—H26	119.1	O4—Cu2—N8	93.70 (15)
C27—C26—H26	119.1	O4—Cu2—N2	86.63 (15)
C26—C27—C22	121.0 (5)	N8—Cu2—N2	176.92 (16)
C26—C27—H27	119.5	O3—Cu2—N7	156.24 (15)
C22—C27—H27	119.5	O4—Cu2—N7	106.46 (15)
C25—C28—H28A	109.5	N8—Cu2—N7	80.59 (16)
C25—C28—H28B	109.5	N2—Cu2—N7	96.37 (16)
H28A—C28—H28B	109.5	O3—Cu2—N1	94.97 (14)
C25—C28—H28C	109.5	O4—Cu2—N1	143.21 (15)
H28A—C28—H28C	109.5	N8—Cu2—N1	102.89 (16)
H28B—C28—H28C	109.5	N2—Cu2—N1	78.49 (16)
C30—C36—H36A	109.5	N7—Cu2—N1	108.50 (14)
C30—C36—H36B	109.5	C56—N1—C52	118.4 (4)
H36A—C36—H36B	109.5	C56—N1—Cu2	128.6 (3)
C30—C36—H36C	109.5	C52—N1—Cu2	112.8 (3)
H36A—C36—H36C	109.5	C47—N2—C51	119.7 (4)
H36B—C36—H36C	109.5	C47—N2—Cu2	123.0 (4)
C31—C30—C29	116.7 (5)	C51—N2—Cu2	117.3 (3)
C31—C30—C36	122.4 (6)	C6—N3—C10	119.8 (5)
C29—C30—C36	120.9 (6)	C6—N3—Cu1	117.7 (3)
C30—C31—C32	123.3 (6)	C10—N3—Cu1	122.0 (4)
C30—C31—H31	118.4	C1—N4—C5	119.2 (5)
C32—C31—H31	118.4	C1—N4—Cu1	129.0 (3)
C33—C32—C31	119.1 (5)	C5—N4—Cu1	111.7 (3)
C33—C32—H32	120.5	C11—N5—C15	118.6 (4)
C31—C32—H32	120.5	C11—N5—Cu1	127.7 (3)
C34—C33—C32	118.3 (5)	C15—N5—Cu1	113.7 (3)
C34—C33—C35	121.2 (4)	C20—N6—C16	119.8 (4)
C32—C33—C35	120.5 (5)	C20—N6—Cu1	124.0 (4)
C29—C34—C33	121.1 (5)	C16—N6—Cu1	116.1 (3)
C29—C34—H34	119.5	C46—N7—C42	117.5 (4)
C33—C34—H34	119.5	C46—N7—Cu2	127.8 (3)
O4—C35—O3	123.7 (4)	C42—N7—Cu2	114.5 (3)
O4—C35—C33	120.0 (4)	C37—N8—C41	118.6 (4)
O3—C35—C33	116.3 (4)	C37—N8—Cu2	125.8 (4)
N8—C37—C38	122.2 (6)	C41—N8—Cu2	115.5 (3)
N8—C37—H37	118.9	C21—O1—Cu1	110.0 (3)
C38—C37—H37	118.9	C35—O3—Cu2	111.0 (3)
C39—C38—C37	119.2 (5)	H2W—O1W—H1W	103.5
C39—C38—H38	120.4		

### Hydrogen-bond geometry (Å, °)

**Table:**

*D*—H···*A*	*D*—H	H···*A*	*D*···*A*	*D*—H···*A*
O1W—H1W···I1^i^	0.82	3.15	3.935 (8)	161
O1W—H2W···I1^ii^	0.82	2.76	3.568 (8)	170

Symmetry codes: (i) −*x*+1, −*y*+1, −*z*+1; (ii) *x*+1, *y*+1, *z*.
